# Multifocal reactive myositis induced by *Klebsiella pneumoniae*

**DOI:** 10.1093/rap/rkab025

**Published:** 2021-04-05

**Authors:** Alexa Escudero Siosi, Hudaifa Al Ani, Nida Chaudhry, Stefen Brady, Antoni Chan

**Affiliations:** 1 Department of Rheumatology, Royal Berkshire Hospital NHS Foundation Trust, Reading; 2 Department of Neurology, John Radcliffe University Hospital, Oxford, UK

Dear Editor, We present the case of a 59-year-old female smoker, with past medical history of chronic kidney disease, congestive cardiac failure, peripheral vascular disease and previous anal cancer, which had been in remission for 9 years following treatment, who presented with sudden-onset left flank and hip pain.

Key message

 • *Klebsiella pneumoniae*-associated reactive myositis may mimic autoimmune myositis; differentiating the two is essential because it affects treatment.

At presentation she was febrile (38.9°C), with a sinus tachycardia (heart rate 113 /min) and blood pressure of 120/72 mmHg. Her abdomen was soft, with mild renal angle tenderness. Her hip pain caused significant difficulty in weight-bearing, with limitation in the range of active movement. Her CRP was 164 mg/l, and estimated glomerular filtration rate decreased from 60 to 45 ml/min. Chest X-ray was normal. Blood cultures were negative. Urine culture was positive for *Klebsiella pneumoniae*. Renal US and CT abdomen were normal. MRI showed increased signal on short-TI inversion recovery sequence (STIR) of the proximal anterior and medial pelvic girdle muscles, consistent with oedema and suggestive of myositis. She was treated with gentamicin 560 mg daily and teicoplanin 960 mg for 9 days. She showed an excellent response, with improvement of inflammatory markers and hip pain.

**Fig. 1.**
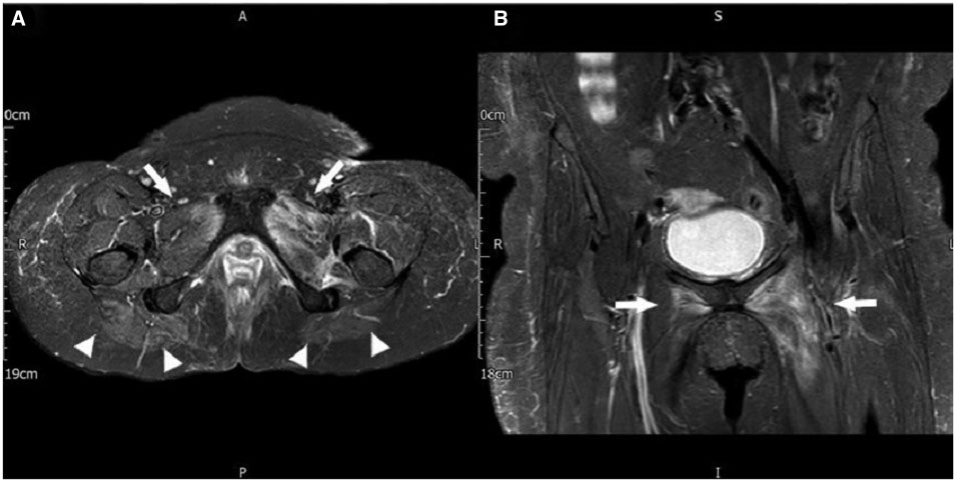
MRI showing multifocal patchy muscle oedema Short tau inversion recovery transverse (STIR _TRA; **A**) and Short-TI Inversion Recovery Coronal (STIR _COR; **B**), Showing multifocal areas of patchy oedema centred within the adductor compartment bilaterally (arrows) and the medial, superior aspect of the gluteal muscles bilaterally (arrowheads).

Four months later, she attended again, with a similar presentation of fever at 38°C and left hip pain. Her results showed high white blood cell count (14.96 × 10^9^ /l), ESR 113 mm/h, CRP 213 mg/l and creatine kinase 301 U/l. HIV, HBV and HCV were negative. Urine cultures and blood cultures were positive for *K. pneumoniae*. An extended myositis immunoblot was negative. Repeat MRI left hip showed changes consistent with multifocal myositis involving the adductor and gluteal compartments bilaterally. There was a small left hip effusion, with pericapsular oedema (Fig. 1). A muscle biopsy of the left adductor compartment was performed, and this did not show features of autoimmune myositis or pyomyositis.

She received gentamicin 120 mg once a day and teicoplanin 900 mg for 8 days and had a good response to this. Her raised creatine kinase normalized after 2 days of treatment to 72 U/l. Her temperature and inflammatory markers returned to normal, and her symptoms of hip pain responded well to a full course of antibiotic therapy.

Further investigation of her recurrent urinary infection was carried out subsequently. CT urinary tract showed a left hydronephrosis and hydroureter. A CT abdomen showed changes of uterine cancer, with free fluid and mesenteric soft tissue stranding within the pelvis impinging on the left ureter and resulting in left-sided hydronephrosis.

In our opinion, infection with *K. pneumoniae* resulted in a reactive myositis on MRI, mimicking an autoimmune myositis. In the latter, the majority of patients have multisystem disease, significantly raised creatine kinase and inflammatory changes on muscle biopsy, which were absent in our case. A paraneoplastic myositis was unlikely in view of the non-progressive nature of the myositis.

The timing and significant response to antimicrobial therapy in conjunction with clinical, imaging and laboratory findings support a diagnosis of infection-associated reactive myositis. She had a left-sided hydronephrosis causing recurrent *Klebsiella* urinary tract infection and leading to myositis. This organism has been reported to cause pyomyositis, but few reactive myositis cases have been described.

Acute infectious myositis is uncommon and most frequently linked to viral respiratory infections, in particular influenza. Isolated attacks of acute bacterial myositis have been reported; however, these are rare and less commonly seen in adults [1].

Bacterial myositis is diffuse muscle infection, without intramuscular abscess formation. The pathogenesis and precise mechanisms by which it causes muscle damage are unclear. It could be direct muscle attack by the pathogen, toxin release or an autoimmune antigenic response [2]. Although a wide variety of microorganisms have been implicated in bacterial myositis, ∼95% of cases are caused by *Staphylococcus aureus* and 1–5% by *Streptococcus pyogenes*. Gram-negative organisms, such as *Enterobacter* and *Escherichia coli*, are rare causes and include *K. pneumoniae*, as in our case [3].

*Klebsiella pneumoniae* infection usually affects immunocompromised patients with neutropenia, HIV and those with diabetes or alcohol abuse [3–5]. Other possible causes of myositis were also explored in our case but were absent, including autoimmune disease, trauma, medications and electrolyte disturbances. In most reported cases, *K. pneumoniae* caused pyomyositis. In our patient, it caused reactive myositis affecting the gluteal muscles bilaterally, which is rarer. However, her immunocompetence could have been affected by her current endometrial cancer in addition to previous anal cancer.

The exact mechanism for the pathogenesis of reactive myositis is uncertain. However, infection is considered to be the inciting event, followed by a deleterious synergistic interaction between the microorganism and pro-inflammatory agents from the host's own defence systems. There is no primary muscle invasion or molecular mimicry with secondary loss of self-tolerance.

Our patient had *K. pneumoniae*-associated reactive myositis, which could be mistaken for autoimmune myositis. It is essential that these are differentiated because it alters management. Treatment of the underlying infection causing reactive myositis is based on appropriate antibiotic therapy, whereas autoimmune myositis is treated with systemic immunosuppression.

*Funding*: No specific funding was received from any funding bodies in the public, commercial or not-for-profit sectors to carry out the work described in this manuscript.

*Disclosure statement*: The authors have declared no conflicts of interest.
